# Thermal Preferences of Cowpea Seed Beetles (*Callosobruchus maculatus*): Effects of Sex and Nuptial Gift Transfers

**DOI:** 10.3390/insects12040310

**Published:** 2021-04-01

**Authors:** Dariusz Krzysztof Małek, Marcin Czarnoleski

**Affiliations:** Institute of Environmental Sciences, Faculty of Biology, Jagiellonian University, 30-387 Kraków, Poland; marcin.czarnoleski@uj.edu.pl

**Keywords:** *Callosobruchus maculatus*, nuptial gifts, temperature, thermal preference, seed beetles, sexual dimorphism, reproductive effort, water balance

## Abstract

**Simple Summary:**

The thermal environment is crucial for organismal functioning, and many cold-blooded organisms, including insects, behaviorally regulate their body temperature. Why do insects inhabit given thermal conditions? We propose that access to water affects thermal preference and that insects with poor access to water inhabit colder environments, which reduces evaporation and preserves water. We studied the seed beetle *Callosobruchus maculatus*, which, as adults, do not drink or eat; however, males provide their mates with sperm, as well as nuptial gifts, including nutrients and water sources. We compared preferred temperatures between males and females that had access to mates or remained unmated and measured the sizes of the transferred gifts. We found that females preferred higher temperatures than males, but these preferences did not change due to mating or the transfer of larger or smaller gifts. It appears that males and females receive and lose certain amounts of water during mating, but they do not alter their thermal preferences according to the amount of water they receive or lose.

**Abstract:**

The thermal environment influences insect performance, but the factors affecting insect thermal preferences are rarely studied. We studied *Callosobruchus maculatus* seed beetles and hypothesized that thermal preferences are influenced by water balance, with individuals with limited water reserves preferring cooler habitats to reduce evaporative water loss. Adult *C. maculatus*, in their flightless morph, do not consume food or water, but a copulating male provides a female with a nuptial gift of ejaculate containing nutrients and water. We hypothesized that gift recipients would prefer warmer habitats than gift donors and that both sexes would plastically adjust their thermal preferences according to the size of the transferred gift. We measured the thermal preference in each sex in individuals that were mated once or were unmated. In the mated group, we measured the sizes of the nuptial gifts and calculated proportional body mass changes in each mate during copulation. Supporting the role of water balance in thermal preference, females preferred warmer habitats than males. Nevertheless, thermal preferences in either sex were not affected by mating status or gift size. It is likely that high rates of mating and gift transfers in *C. maculatus* living under natural conditions promoted the evolution of constitutive sex-dependent thermal preferences.

## 1. Introduction

The thermal environment has profound and complex consequences on ecological and evolutionary processes [1], including alterations in resource supply and demand [2,3], resource expenditure [4,5,6], multitrophic interactions [7], and mortality rates [8]. In ectotherms, thermal conditions are the key environmental element that directly dictates body temperature and thus physiological rates, shaping organismal performance in the environment and ultimately affecting evolutionary fitness, e.g., in [9]. Certainly, the adaptive value of different life strategies in a given thermal environment depends greatly on the thermal sensitivity of processes that determine organismal performance and the capacity to regulate body temperature [1]. Having limited physiological thermoregulation ability [10], most ectotherms rely on behavioral regulation of body temperature [11], which has clear adaptive value because it helps to achieve desired physiological states [12]. However, inhabiting a thermal environment does not necessarily ensure a body temperature that is universally optimal for all physiological processes and thus similarly impacts all fitness components. For this reason, using different measures of physiological performance may lead to inconsistent conclusions about thermal optima, even in the same species (see, for example, [13,14]). Ultimately, given the life history principles [15] and predicted impacts of the thermal environment on resource allocation [16], organisms should evolve toward a preference for thermal environments that help to maintain an optimal balance between maximizing physiological capacity to produce new tissue (own and offspring) and minimizing the risk of individual and ecological mortality. This might explain why thermal preferences have been shown to vary in association with a wide range of factors, including oxygen conditions [17], body size [18], and interactions with other species [19], and that preferences among the same species can differ between laboratory and natural conditions [20]. Considering broad evolutionary and ecological contexts ensures an improved understanding of patterns of thermal preferences.

In some species, thermal preferences have been reported to differ between sexes [21,22] and change following mating [23]. Although we still poorly understand how this variance is associated with fitness effects, we expect that reproductive investments promote thermal preferences toward conditions amenable to the physiological demands imposed by reproduction. Accordingly, we performed an experimental study in the cowpea seed beetle *Callosobruchus maculatus*, exploring connections between resources transferred between mating partners and the thermal conditions selected by each sex. We took advantage of the reproductive biology of *C. maculatus*, which involves the transfer of a so-called nuptial gift during mating in the form of ejaculate [24]. We hypothesized that this element of reproduction, costly to one partner (male) but beneficial to the other (female), should affect thermal preferences in a sex-specific way. Nuptial gifts have originated independently in many different taxa and are defined as “materials (beyond the obligatory gametes) provided by a donor to a recipient during courtship or copulation in order to improve donor fitness” [25]. In most species, including *C. maculatus*, females are the recipients, while males are the donors of nuptial gifts (e.g., [25], but see [26]). Nuptial gifts can take various forms, as classified by Lewis et al. [25], but the endogenous gifts provided by the donor have great influence on the physiology of both the donor and the recipient. Typically, nuptial gifts are regarded as an extra source of nutrients and energy [25,27], which is also commonly invoked for *C. maculatus* [28,29,30,31]. Earlier studies in *C. maculatus* showed that reproductive activity reduces the lifespan of males [32]; at the same time, females benefit from mating with either virgin males [32] or mating multiple times during their lifespan [28]. There is evidence that the size of a gift has quantitative effects on the fitness of *C. maculatus*, with larger gifts corresponding to better survival among recipient females but worse survival among donor males [33]. Importantly, the ejaculate of *C. maculatus* has been shown to provide females with water [34,35], which suggests another function of ejaculate: Water supply for females. Certainly, given that mitochondrial respiration produces not only ATP, but also metabolic water [36], the organic compounds in nuptial gifts can be safely regarded as water sources, which are either gained (recipient) or lost (donor) during gift transfer between mates. Here, we consider that ectotherms exposed to warm environments face not only increased metabolic demand, but also increased evaporative water loss [1,37]. In effect, nuptial gifts could aid in balancing increased water demands in hot or arid environments to some extent. Focusing specifically on *C. maculatus*, we predicted that gift recipients (females) would select higher temperatures than gift donors (males). We further considered that the thermal preferences of each sex might undergo plastic changes after the actual transfer of resources via gifts, shifting following the mating of virgin individuals toward warmer sites in females or cooler sites in males. Moreover, we explored whether these two responses further depended on the amount of resources transferred in the ejaculate.

It is important to emphasize here that *C. maculatus* is uniquely relevant for addressing adaptations to a desiccation risk. The species originates from seasonal dry environments in West Africa, but now it occurs globally as a pest of stored legumes [38,39], and its present form and the evolutionary past are tightly linked to the history of crop domestication. The cowpea (*Vigna unguiculata* L. Walp.), the original host plant of *C. maculatus*, is considered as one of the oldest (c.a. 4000 years) human crops [40]. It is telling that in semi-natural conditions in Africa (growing legumes in the field and stored legume seeds), *C. maculatus* produces two distinct life forms each year: A mobile flight morph that lays eggs directly to seed pods of growing plants during the rainy season, and a sedentary flightless morph that develops in seeds gathered by humans and stored in dry places [41,42]. The morphs are induced during a post-embryonic development by environmental cues, including temperature, seed water content, and larval density [43,44,45]. The flightless morphs prevail in the life cycle of *C. maculatus*, with up to five subsequent generations of the flightless morph vs. only one generation of the flight morph per year [41,46,47,48]. Importantly, controlled laboratory conditions commonly maintain *C. maculatus* indefinitely in its flightless morph, mimicking the semi-natural conditions in the legume storage. The flightless morph has low capacity to move long distances and to leave seed stores in search for food and water in the environment. Not surprisingly, the flightless morph does not require feeding or access to water to complete its life cycle (aphagia) [49]. To our advantage, this aspect of biology infers especially strong resource and water limitations in reproducing adults. By contrast, the flight morph of *C. maculatus* likely feeds on non-host pollen and returns for oviposition, as suggested by the evidence for other bruchids [50,51]. 

## 2. Materials and Methods

The *C. maculatus* beetles used in this study originated from a laboratory stock culture maintained at the Institute of Environmental Sciences Jagiellonian University in Krakow, Poland. The culture was established from commercially available insects from the Invertebrate Supply Unit, Fera Science Limited, London, United Kingdom, and was maintained and reared on a standard medium of cowpea seeds (*Vigna unguiculata*) under 12 h dark/12 h light at 27 °C in thermal cabinets (Pol-Eko-Aparatura sp.j., Wodzisław Śląski, Poland). 

To obtain insects for the experiment, we collected approximately 300 bean seeds from the stock culture, each with a single *C. maculatus* egg. To eliminate the potential effects of nutrient limitations, at this stage, we excluded the smallest seeds (less than approximately 150 mg). While multiple *C. maculatus* larvae can develop inside a single cowpea seed, our previous results suggested that the effects of seed size variation on adult phenotypes cannot be ignored, even if a single larva develops inside a seed [33]. Each egg-bearing seed was placed in a separate Eppendorf tube with a perforated lid. The tubes were checked every 12 h for adult emergence, which allowed us to control the age of adults entering our experiment. After emergence, beetles were sexed, and each male and female were randomly assigned to one of two experimental groups, according to mating experience: The mated group, consisting of virgin individuals that were allowed to mate under controlled conditions to allow gift transfer, or the nonmated group, consisting of virgin individuals that were not allowed to mate and exchange gifts. For logistical reasons (see below), we were able to measure thermal preferences in four animals daily. Given our aim to control the age of animals entering our experiment (see below), we had to spread the measurements over several days, discarding some animals, if we were not able to involve them in the measurements at the right time. Ultimately, we measured thermal preferences in 115 beetles, including 63 males (38 nonmated and 25 mated) and 53 females (29 nonmated and 24 mated).

Approximately 24 h (±12 h) after emergence from the bean seeds, each beetle was weighed to the nearest 0.001 mg using a microbalance (Mettler-Toledo XP26, Mettler-Toledo GmbH, Laboratory & Weighing Technologies, CH-8606 Greifensee, Switzerland). In the mated group, males and females were paired in Eppendorf tubes to allow a single copulation to take place. After copulation, males were weighed again. Following previous research (see, for example, [52]), we calculated the mass of the nuptial gift according to the decrease in body mass in a male after copulation. This information was used to estimate the amount of resources lost by a male and the amount of resources gained by their female partner. In all cases when mating did not result in a measurable loss of body mass in males, the size of the nuptial gift was considered to be equal to zero. For standardization between the mated and nonmated groups, individuals in the nonmated group were exposed to conditions similar to those in the mated group during mating and body mass measurement (e.g., both groups were removed from the thermal cabinet and exposed to mating and measuring conditions for the same amount of time). After these procedures, all beetles were placed back under the experimental conditions, and after 1 h, they were subjected to tests for thermal preferences.

**Figure 1:**
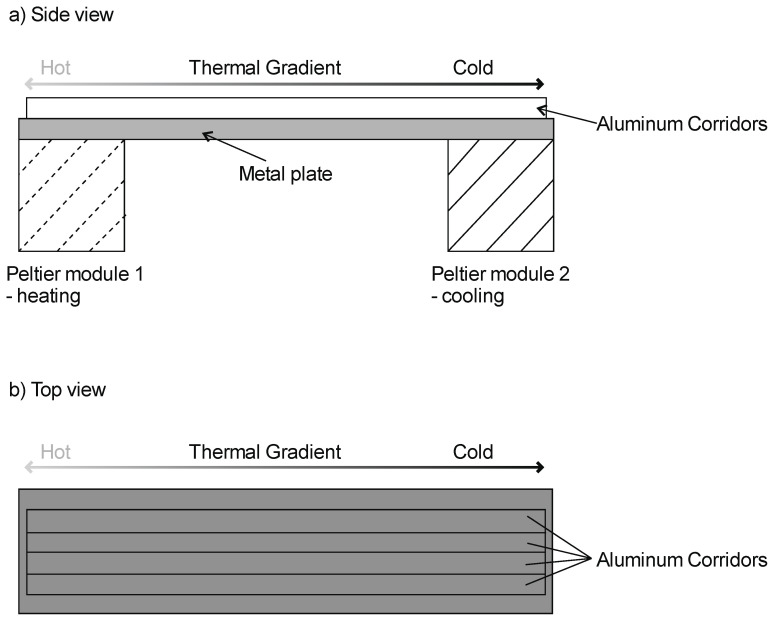
Equipment used to generate the thermal gradient for measuring *Callosobruchus maculatus* beetles’ thermal preferences. Individual beetles were placed in each corridor, and the corridors were covered with transparent plastic wrap. After 75 min, a thin thermocouple was used to measure the temperature of the aluminum substrate next to the sitting beetle.

Thermal preferences were measured in a modified setup based on that in a study by Antoł et al. [17] on the thermal behavior of woodlice. It involved an apparatus (Figure 1) that created a thermal gradient along a 1 m long aluminum bar (the arena). Along the length of the arena, we placed four aluminum U-shaped profiles, which served as partitions for the independent testing of four animals at a time. Each corridor was covered with transparent plastic wrap, which prevented air exchange with the environment and allowed us to visualize the position of the tested animal in the corridor. On each side, the arena was in contact with a Peltier module, which was set to either heating (one side) or cooling (the other side). The apparatus was placed in a climatic room set to 26 °C. The temperatures on the surfaces of the corridors were measured to the nearest 0.05 °C with a DELTA HD2128.1 A thermometer (Delta Ohm S.R.L., Selvazzano, Italy) connected to a thin thermocouple (1 mm in diameter). The thermocouple was exposed to the corridors through a minute hole, generated by the gentle puncturing of the stretch wrap in each corridor. Prior to the experiment, the temperatures were measured in three places: At both ends and at the center of each corridor. This was repeated five times for each corridor at 1 h intervals. Subsequently, the average temperatures were calculated for each location in the corridor. Based on these data, we determined that the insects experienced surface temperatures ranging from 16 to 36 °C, with a ~0.2 °C per cm gradual change. Following Dillon et al. [53], we avoided directional light during the experiment using a set of dim fluorescent lights mounted to the ceiling, which uniformly distributed light above the arena with the thermal gradient. Before each measurement, corridors were cleaned with tissue to reduce potential effects of chemical substances left by beetles on consecutive measurements, and insects were individually placed in the corridors at positions close to the warmest end of the gradient. Then, the corridors were immediately covered with plastic wrap and the insects were allowed to move freely for 75 min in the corridors and were able to encounter the whole thermal gradient. Our preliminary tests showed that after 75 min, most beetles had settled in place, and this was used as an indication of microsite preference. To measure the thermal conditions in the chosen microhabitat (to the nearest 0.05 °C), we perforated the plastic wrap with a thin thermocouple connected to our DELTA thermometer, touching the aluminum substrate with the tip next to the sitting animal. 

Statistical analysis was performed with Statistica 13 software (TIBCO Software Inc., Palo Alto, CA, USA). Before the analysis, preferred temperature data were cube-transformed to achieve a normal distribution; the remaining data were analyzed without transformation because they met the assumption of a normal distribution. First, we compared body mass between males and females with a general linear model (GLM). This analysis assessed data of the initial body mass (before mating) of all the insects involved in the study (mated and nonmated). Addressing whether thermal preferences changed with sex and mating status, we analyzed data on preferred temperatures with a GLM that included sex (male vs. female) and mating status (mated vs. nonmated) as fixed predictors. The model also included a sex × mating status interaction, which tested our hypothesis that mating shifted the thermal preferences of males and females in different directions. This model considered the data from each of the mated partners as two independent observations. In the next step, we constructed a similar GLM for preferred temperature, but with an additional predictor—an individual’s body mass (initial value before mating)—as a continuous variable. This model also included sex × mating status interactions. By analyzing sex differences in thermal preferences using these two models, we explored to what extent the potential sex effects on thermal preferences might be attributed to body mass differences between sexes. Addressing whether the size of the nuptial gift affected the thermal preference, we analyzed preferred temperatures with other GLMs, focusing on only mated animals. These analyses were performed separately for each sex. The models included relative nuptial gift size as a numeric fixed predictor, calculated as a percentage of body mass of the donor/recipient. To meet the assumptions of parametric tests, prior to the analysis, the relative gift size was transformed by calculating its arcsine square root [54]. The complete dataset used is available in the supplementary materials (Table S1). 

## 3. Results

**Figure 2:**
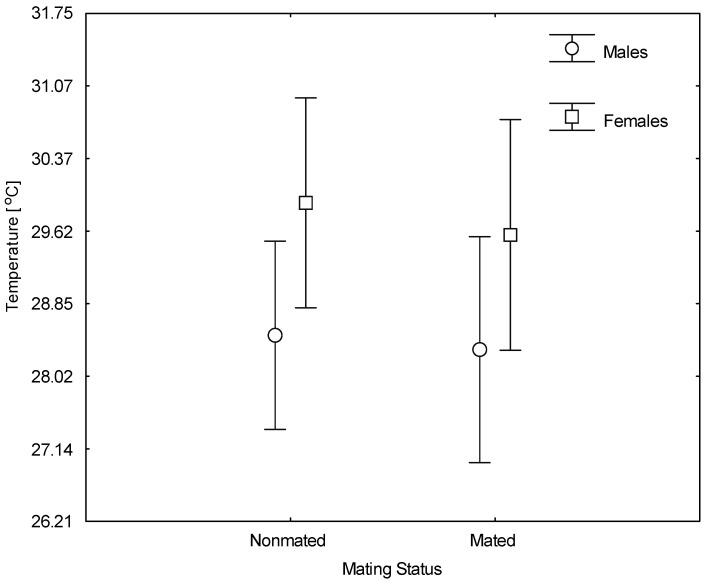
The effect of sex and mating status (nonmated—virgin individuals; mated—virgin individuals that were allowed to mate once) on the preferred temperature of *Callosobruchus maculatus beetles.* Vertical bars denote 95% confidence intervals (see Table 1). In the statistical model, the temperature data were cube-transformed, but they were back-transformed for presentation purposes. Note that only the effects of sex were significant.

Before mating (all virgin beetles, including beetles that were subsequently mated), female *C. maculatus* were heavier than male *C. maculatus* (F = 512.65, *p* < 0.001). In the thermal preference tests, beetles were found in microsites that spanned a wide range of temperatures, from 19.0 to 34.9 °C. Our GLM for all experimental beetles analyzed together (Table 1a) showed that on average, females occupied sites that were warmer than the sites occupied by males (29.8 vs. 28.4 °C; *p* = 0.024; Table 1a and Figure 2). Mating status (mated vs. nonmated) did not change these preferences (*p* = 0.669; Table 1a). There was also no significant interaction between sex and mating status (*p* = 0.870; Table 1a and Figure 2), indicating consistent sex differences in thermal preference, which was not affected by mating. After taking the initial body mass of the animals into account, the GLM (Table 1b) showed no significant effect of sex (*p* = 0.107; Table 1b). This model also did not show effects of any other factors, including mating status (*p* = 0.603), body mass (*p* = 0.489), or sex × mating status interaction (*p* = 0.747). Focusing on only mated individuals, the GLM showed no significant effects of variations in nuptial gift size on thermal preferences in either males (*p* = 0.859, Table 2a) or females (*p* = 0.377; Table 2b).

## 4. Discussion

In our experiment, *C. maculatus* beetles were provided a choice of thermal microsites spanning approximately 20 °C. The results showed that they were not distributed randomly along the thermal gradient. To a large extent, temperature preferences in the occupied microenvironment were attributable to the sex of the beetles, with females preferring warmer microsites than males, irrespective of whether the animals were allowed to mate before the thermal preference assay. To the best of our knowledge, these findings represent the first published evidence of sex-related thermal preferences in *C. maculatus*, but we stress that what fitness benefits *C. maculatus* females gain from inhabiting warm environments remains unknown. The detected pattern is consistent with our hypothesis that at least some resources in male ejaculate may be utilized by female recipients as an external source of water. Consequently, females of this species should become less water-limited than males, and thus able to inhabit warmer microhabitats with an increased risk of desiccation. This scenario seems especially probable in the light of the alteration of the two distinct morphs of *C. maculatus* under semi-natural conditions. Inevitably, multiple generations of the sedentary flightless morph that occur consecutively in a single seed storage facility face severe limitations in water and food supply [41,47,48]. Certainly, further research should resolve whether the nuptial gifts of *C. maculatus* contain actual water and/or energetic supplies that are used as a source of metabolic water, though we note that these two alternative scenarios are not mutually exclusive. 

It is tempting to extrapolate our findings on a large evolutionary scale, suggesting that the evolutionary origin of nuptial gifts might have been followed by sex-specific shifts in thermal preferences toward warmer sites for gift recipients and cooler sites for gift donors. Unfortunately, it appears that sex differences in thermal preference have rarely been studied, and among the studies that have been performed, the evidence is biased toward some groups of animals, such as reptiles (e.g., [55,56,57,58], but see [59,60]). It is difficult to find relevant evidence in species who provide nuptial gifts. Oral nuptial gifts are common among crickets (se [61]), but sex-related thermal preferences have not been studied in this group. Interestingly, females of the cricket *Gryllodes sigillatus* increase mating frequency with temperature [62], which can indicate effects of thermal dependence of physiology, but it also suggests that females exposed to high rates of evaporative water loss in hot environments increase mating frequency to obtain additional nuptial gifts and, thus, additional water supplies. Among species of fruit flies, *Drosophila subobscura* displays a type of nuptial feeding (oral nuptial gift), but to the best of our knowledge, thermal preferences in this species have been studied in only females and in a different context [63]. Apart from the importance of nuptial gifts in energy budgets (as defined in [25]), nuptial gifts, both oral and genital, may serve other functions, which can complicate studies aimed at analyzing the effects of nuptial gifts on balancing water supply across thermal gradients. For example, nuptial gifts have been shown to contain immunostimulatory and antibiotic components [64], specialized defensive substances [65], and substances that manipulate recipient behavior [66]. Studies aimed at exploring relationships between sex-specific thermal preferences and nuptial gifts are additionally complicated by a multitude of factors that should be considered potential drivers of thermal preferences and reproductive investments, e.g., visible even in a single group of closely related *Drosophila* species. For example, in *D. melanogaster* and *D. simulans*, preferred temperature differs by sex, but female thermal preferences are also dependent on the rearing temperature, with those reared at cooler temperatures showing a preference for warmer temperatures [67]. At the same time, in *D. melanogaster*, many seminal fluid substances change the behavior of females following mating [66], and in *D. simulans*, while there is no direct information about nuptial gifts, females tend to have greater lifetime reproductive success but die at a younger aged if mated more than once [68], suggesting that some seminal substances modify female behavior and physiology. There are also other costly reproductive mechanisms in other *Drosophila* species, such as ejaculate with highly costly giant sperm in *D. littondis* and *D. hydei* [69] of the *D. virillis* species group, which do not seem to provide any nutritional benefits to females [70]. However, female *D. virilis* generally show a preference toward warmer conditions [71]. Among other insects that provide nuptial gifts, females of some species, such as the hemipteran *Rhodnius prolixus* [72], show a preference toward warmer conditions, while other species, such as *Pteronidea melanaspis* sawflies, show contrasting sex differences [59]; furthermore, some other species, such as *D. immigrans*, do not show any sex difference in thermal preference [71]. Among other animal groups, there are examples of females preferring warmer sites (e.g., *Crotaphytus collaris* lizards, [73]; yellow-margined box turtles *Cuora flavomarginata*, [57]; mice, [60]), males preferring warmer sites (e.g., lobsters [21]; other lizard species [22]), and no apparent sex differences in preferred temperature (e.g., Aesculapian snakes and green whip snakes [74]). Interestingly, a study on common rough woodlice showed no sex differences in thermal preference, but decreased oxygen availability was found to reduce preferred temperatures in both sexes [17].

Similar to many other insect species (see [75]), *C. maculatus* shows body mass dimorphism, with females being heavier than males (see, for example, [76] and our data). Interestingly, our results showed that thermal preference was significantly affected by sex only when data were analyzed without considering beetle body mass, although we found that body mass was not a significant predictor of thermal preference according to the model. Overall, our data showed that the preference of *C. maculatus* toward warmer conditions was characteristic of females, which were also the larger sex. Interestingly, this result agrees with the hypothesis that the risk of desiccation is involved in the selection of the thermal environment by ectotherms. This is because a large body mass is associated with a relatively small body surface area, which would make warm habitats with high rates of evaporation less demanding for large-bodied females than for small-bodied males. Evidence addressing this hypothesis is scarce. Morita et al. [18] showed that larger fish prefer colder water temperatures, but body size differences are inherently linked to age effects (larger fish are older), and importantly, aquatic animals are not useful in addressing the role of water loss in size differences in thermal preferences of terrestrial organisms. Some studies in lizards showed no significant influence of body size on thermal preference (e.g., [77]). Additionally, under field conditions, the sexes often differ significantly in their behavior, and this may result in inhabiting different thermal microenvironments (for example, males actively search for mates and females search for oviposition sites). In such cases, any physical constraints imposed by sex-specific body size may be offset by differences among microenvironments [78]. According to our results, body mass itself should not be considered a strong predictor of thermal preference.

According to our calculations, the largest ejaculate of *C. maculatus* resulted in an 8% loss in male body mass and a 6% gain in female body mass. These data indicate that during a single mating event between two virgin individuals, males lose a large fraction of their body mass, while females gain a large amount of body mass. If the thermal preference of *C. maculatus* depends on the water supply in ejaculate, then mating and the actual size of the transferred nuptial gift should result in a shift in thermal preference toward warmer temperatures in gift recipients and cooler temperatures in gift donors. In contrast to this expectation, neither the mating of virgin individuals nor the mass of the transferred ejaculate had measurable effects on thermal preferences in either sex of *C. maculatus*. Nevertheless, it is likely that natural selection favors constitutive sex differences in the thermal preferences of *C. maculatus*, driven by the expected rate of mating and thus the intensity of transfers of nuptial gifts from males to females. Plastic adjustments in thermal preference in response to mating should not result in a selective advantage, if under ecologically relevant conditions, emerging adults do not experience prolonged periods without mating, which is likely the case of flightless morphs of *C. maculatus*, which forms dense populations in seed supplies managed by humans and have very limited opportunities for dispersal. Certainly, it would be premature to completely abandon the idea that the actual transfer of a gift induces a change in the thermal preference of *C. maculatus* because the ability to detect such effects can depend on the experimental design. For example, in our experiment, thermal preferences were studied immediately after the first mating of virgin individuals, and mates were not exposed to an egg-laying medium (bean seeds). Moreover, we do not know how quickly and with reference to what cues mating partners might adjust their thermal preference according to the size of the ejaculate. Additionally, it would be important to consider whether the involvement of egg laying in females and the involvement of searching for mates in males play additional roles in the thermal preferences of *C. maculatus*. In *D. melanogaster*, females show strong preferences for oviposition site temperatures [79]. In *Lacerta vivipara*, females change their thermal preferences according to the different phases of gestation [23]. By selecting thermal conditions during egg production, ovipositing ectothermic females affect their own physiological capacity to produce eggs and the developmental conditions for their progeny. In insects, egg production is known to be highly dependent on environmental temperature [80], and in *C. maculatus*, higher temperatures have been demonstrated to positively affect the number of eggs laid by females, as well as egg hatchability [81]. Developmental temperatures strongly affect fitness components in *C. maculatus* [82,83] and other insects (e.g., [84,85]). Thermal conditions have also been shown to affect the longevity of *C. maculatus* [86].

## 5. Conclusions

Overall, we demonstrated that the beetle *C. maculatus* exhibited sex differences in preferred thermal conditions, with large-bodied females selecting warmer sites and small-bodied males selecting cooler sites. This finding supports the idea that nuptial gifts may be important in supplying water to mating partners, which is gained in females but lost in males. It remains to be resolved whether the nuptial gifts of *C. maculatus* directly contain water or organic compounds that are utilized for metabolic water production. We failed to demonstrate that *C. maculatus* plastically adjusts their thermal preference according to the actual transfer of a nuptial gift, which weakens our conclusions about the role of nuptial gifts in thermal preference. Nevertheless, we envision that high rates of mating and, thus, gift transfers under conditions met in dry legume seed storage promoted the evolution of flightless beetle forms with constitutive sex differences in thermal preferences rather than plastically changing thermal preferences. We conclude that future studies should consider the role of nuptial gifts as sources of water to better understand the thermal biology of *C. maculatus*. Nevertheless, we acknowledge the need to identify other factors that can simultaneously shape intersexual differences in thermal preference, such as the thermal requirements for egg production and offspring development. 

## Figures and Tables

**Table 1:** The results of two general linear models of preferred temperatures of *Callosobruchus maculatus* beetles, without and with body mass as a covariate. Mating status represents either nonmated beetles (virgin individuals) or mated beetles (virgin individuals that were allowed to mate once).

	Factor	Df	F	*p*
(a) Body mass excluded	Sex	1	5.218	0.024
	Mating Status	1	0.184	0.669
	Sex × Mating Status	1	0.027	0.870
	Error	110		
(b) Body mass included	Sex	1	0.038	0.845
	Mating Status	1	0.107	0.744
	Body mass	1	0.528	0.469
	Sex × Mating Status	1	0.002	0.967
	Error	108		

**Table 2:** The results of the general linear model analysis of preferred temperatures of mated *Callosobruchus maculatus* beetles (individuals allowed to mate once). Analyses for each sex were performed independently. Nuptial gift size was expressed in relative values as the proportion of initial body mass gained by females or lost by males following a single mating.

	Factor	Df	F	*p*
(a) Males	Nuptial Gift Size	1	0.179	0.676
	Error	23		
(b) Females	Nuptial Gift Size	1	2.013	0.171
	Error	21		

## Data Availability

The complete dataset is available in the Supplementary Materials.

## Supplementary Materials

The following are available online at https://www.mdpi.com/article/10.3390/insects12040310/s1, Table S1: Complete dataset.
